# A New Danger to the Race

**Published:** 1883-06

**Authors:** 


### Editorial.

### A NEW DANGER TO THE RACE.

The tendency of medical advancement and discovery is evidently in a direction antagonistic to the great principle of Darwinism, the survival of the fittest. Hence the assiduous cultivation of almost endless races and generations of microphites by Pasteur and Koch.

One of the chief objects of attraction at the great Cotton Exposition of Atlanta was the wonderful chicken incubator, by which that useful bird can be got up to a frying size in an incredible short time, while the fathers and mothers having nothing to do but prepare more eggs for the machine.

And now Dr. Tavernier, a learned and ingenious physician, has come to the front with a baby incubator more wonderful than that of the chicks. If this last invention proves equal to its claims, as set forth by the *Sanitary News*, one or the other of two things must be done, or the old philosopher Malthus, of political ethics fame, will burst indignant from his tomb.

Either this child incubator must be hedged in by legislative enactment, or another discovery must be quickly made supplementary to the chicken hatcher—a process by which the cereal grains may be artificially and indefinitely propagated.

The following is Dr. Tavernier's process, as given in the *Sanitary News*:

"The report of some remarkable experiments in so-called artificial child incubation comes from France. The *Glasgow Mail* says that the immense success which has attended the artificial incubation of children of France recently attracted the attention of Dr. Tavernier, a learned and ingenious physician. He was attached to a hospital for foundlings, and was annoyed at the large number of foundlings who died within the first six months of their life. The majority of those admitted to the hospital were weak and sickly, and he resolved to try what 'artificial incubation' would accomplish if applied to infants. The doctor constructed a child incubator on precisely the model of the ordinary chicken incubator. It was a box covered with a glass slide, furnished with a soft woolen bed, and kept at the temperature of eighty-six degrees Fahrenheit, by the aid of hot water. He selected as the subject of his first experiment a miserably-made infant, one that had come into the world at an injudiciously early period. This infant was placed in the incubator, provided with a nursing bottle, and kept in a dark room. To the surprise of the doctor, it ceased to cry on the second day it was placed in the incubator, and although it had previously been a preternaturally sleepless child, it sank into a deep and quiet sleep. The child remained in the incubator for about eight weeks, during which time it never once cried, and never remained awake except when taking nourishment. It grew rapidly, and when, at the expiration of sixty days, it was removed from the incubator, it presented the appearance of a healthy infant of at least a year old. Delighted with the success of the experiment, Dr. Tavernier next selected an ordinary six-months-old infant addicted to the usual pains and colic, and exhibiting the usual fretfulness of French infants. This child conducted itself while in the incubator precisely as its predecessor had done. It never cried; it spent its whole time in sleep, and it grew as if it had made up its mind to embrace the career of a professional giant. After a six weeks' stay in the incubator it was removed and weighed. It had become so strong and healthy that it resembled a child three years old, and it could actually walk when holding on to a convenient piece of furniture. These two experiments satisfied Dr. Tavernier of the vast advantages of artificial child incubation. He immediately proceeded, with the permission of the authorities of the hospital, to construct an incubator of the capacity of 400 infants, and in it he placed every one of the 300 infants who were in the hospital on the 10th day of February last. With the exception of one who died of congenital hydrocephalus, and another who was claimed by its repentant parents, the infants were kept continuously in the incubator for six months, when they were removed in consequence of having outgrown their narrow bed. The result will seem almost incredible to persons who are unfamillar with the reputation of Dr. Tavernier, and have not seen the report made to the French government on the subject by a select committee of twelve. The average of the infants last February was eight months and three days—the youngest being less than twelve hours old, and the oldest not more than eleven months. Their average weight was 16 pounds, only one of the entire 360 having attained a weight of 32 pounds. At the end of six months of artificial incubation the average weight of each infant was 24 pounds, and there was not one who would not be supposed by a casual observer to be at least three years old. In other words, six months of artificial incubation did as much in the way of developing Dr. Tavernier's foundlings as three years of ordinary life would have done. The infants were strong and healthy as well as big; they walked within a week of leaving the incubator, and most of them have since learned to talk. These results surpassed Dr. Taverneir's most enthusiastic expectations, and there can be no doubt that his system of artificial child incubation will be adopted not only in every child's hospital in France, but in every private family throughout the civilized world."

